# 供者纯化CD34^+^细胞输注治疗再生障碍性贫血单倍体造血干细胞移植后造血重建不良11例临床分析

**DOI:** 10.3760/cma.j.cn121090-20240902-00331

**Published:** 2025-07

**Authors:** 云 何, 郑丽 徐, 欢 陈, 瑶 陈, 婷婷 韩, 圆圆 张, 萌 吕, 晓冬 莫, 晨华 闫, 昱 王, 于谦 孙, 晓辉 张, 晓军 黄, 兰平 许

**Affiliations:** 北京大学人民医院血液科，北京大学血液病研究所，国家血液系统疾病临床医学研究中心，造血干细胞移植治疗血液病北京市重点实验室，北京 100044 Peking University People's Hospital, Peking University Institute of Hematology, National Clinical Research Center for Hematologic Disease, Beijing 100044, China

**Keywords:** 贫血，再生障碍性, 纯化供者CD34^+^细胞, 单倍体造血干细胞移植, 造血重建不良, Anemia, aplastic, Donor purified CD34^+^ stem cells boost, Haploid hematopoietic stem cell transplantation, Poor hematopoietic reconstruction

## Abstract

**目的:**

分析供者纯化CD34^+^细胞输注治疗再生障碍性贫血（AA）患者单倍体造血干细胞移植（haplo-HSCT）后造血重建不良（PHR）的安全性和有效性。

**方法:**

纳入2019年1月1日至2024年3月31日在北京大学人民医院接受供者CD34^+^细胞输注的11例haplo-HSCT后发生PHR的AA患者，对其输注CD34^+^细胞后造血恢复、移植物抗宿主病（GVHD）发生及预后指标进行回顾性分析。

**结果:**

11例PHR患者中，持续性血小板减少（PT）2例，原发性植入功能不良（PGF）1例，继发性PGF 8例。诊断PHR的中位时间为移植后110（60～330）d，输注纯化CD34^+^细胞的中位时间为移植后194（125～456）d。以输注CD34^+^细胞后180 d为观察点进行疗效评估，2例PT患者分别在CD34^+^细胞输注后22 d、13 d获得血小板恢复，其余9例PGF患者中6例获得完全造血恢复（CHR），1例评估为部分造血恢复（PR），2例评估为无效（NR）。6例获得CHR患者的中性粒细胞计数中位恢复时间为19（8～158）d，HGB中位恢复时间为32.5（13～158）d，血小板计数中位恢复时间为31.5（7～171）d。供者纯化CD34^+^细胞输注后慢性GVHD发生率为18.2％，未发生急性GVHD。中位随访时间为614（153～1 765）d，11例患者中10例存活。

**结论:**

供者纯化CD34^+^细胞输注是治疗AA患者haplo-HSCT后PHR的有效方法。

## Abstract (en)

**Objective:**

To evaluate the safety and efficacy of donor-purified CD34^+^ stem cell boosts in patients with poor hematopoietic reconstruction（PHR）after haploid hematopoietic stem cell transplantation（haplo-HSCT）for aplastic anemia（AA）.

**Methods:**

A retrospective analysis was conducted on 11 patients with AA and PHR who underwent haplo-HSCT and received donor-purified CD34^+^ stem cell boosts at Peking University People's Hospital. Recovery of blood cell counts, incidence of graft-versus-host disease（GVHD）, and overall survival（OS）were assessed.

**Results:**

Of the 11 patients with PHR, two were diagnosed with prolonged isolated thrombocytopenia（PT）, one was primary poor graft function（PGF）, and eight were diagnosed with secondary PGF. The median time to PHR diagnosis was 110 days（range: 60–330 days）, and the median interval from transplantation to purified CD34^+^ hematopoietic stem cell infusion was 194 days（range: 125–456 days）. The two patients with PT achieved complete platelet recovery at 22 and 13 days after CD34^+^ stem cell infusion, respectively. Among the remaining nine patients with PGF, six achieved complete hematopoietic recovery, with a median absolute neutrophil count recovery time of 19 days（8–158 days）, HGB recovery time of 32.5 days（range: 13–158 days）, and platelet recovery time of 31.5 days（range: 7–171 days）. The incidence of chronic GVHD after infusion was 18.2％, with no cases of acute GVHD observed. The OS rate was 90.9％（10/11）in the 11 patients, with a median follow-up of 614 days（range: 153–1 765 days）.

**Conclusion:**

Donor-purified CD34^+^ stem cell boost may be an effective therapeutic strategy for PHR in patients with AA after haplo-HSCT.

再生障碍性贫血（AA）是一种骨髓造血衰竭综合征，异基因造血干细胞移植（allo-HSCT）是治疗重型/极重型AA（SAA/VSAA）及输血依赖型非重型AA（TD-NSAA）的主要治疗方案。移植后造血干细胞完全嵌合但血象未能恢复，称之为造血重建不良（poor hematopoietic reconstitution, PHR），包括植入功能不良（poor graft function, PGF）和持续性血小板减少（prolonged isolated thrombocytopenia, PT）。allo-HSCT后PGF的发生率为5％～27％[1]，原发性PT的发生率为10.1％[2]，AA患者allo-HSCT后PGF的发生率为4.62％～15％[3]–[5]。PGF及PT均可导致非复发死亡增加而严重影响移植预后[2],[6]–[7]。

PHR的发生机制尚未阐明，可能的发病机制包括移植前铁过载、巨细胞病毒（CMV）感染、移植物抗宿主反应病（GVHD）和CD34^+^细胞数量或质量受损[8]–[12]。

目前SAA患者移植后PHR的治疗方法包括G-CSF、输血、雄激素、糖皮质激素、血小板生成素受体激动剂（TPO-RA）等，效果均欠佳。理论上二次造血干细胞移植可能快速重建造血，但限于各种情况，二次移植并不能适用于所有患者。

国外文献报道供者纯化CD34^+^细胞输注可促进恶性血液病患者allo-HSCT后造血干/祖细胞重建，有效率为58.3％～100％[13]–[22]。本研究对我院近年来接受供者纯化CD34^+^细胞输注治疗的单倍体造血干细胞移植（单倍体移植）后PHR的AA患者进行回顾性分析。

## 病例与方法

一、病例

本研究为回顾性分析，纳入2019年1月1日至2024年3月31日期间在北京大学人民医院接受供者纯化CD34^+^细胞输注的11例单倍体移植后PHR的AA患者。所有患者均接受过糖皮质激素、血小板生成素（TPO）/TPO-RA等治疗，未获明显疗效。

二、预处理方案及GVHD预防

预处理方案：①白消安（Bu）/环磷酰胺（Cy）+抗胸腺细胞球蛋白（ATG）：Bu 3.2 mg·kg^−1^·d^−1^，−8 d～−7 d；Cy 50 mg·kg^−1^·d^−1^，−5 d～−2 d；兔抗人胸腺细胞免疫球蛋白（rATG）2.5 mg·kg^−1^·d^−1^，−5 d～−2 d。②Bu/Cy+氟达拉滨（Flu）+ATG：Bu 3.2 mg·kg^−1^·d^−1^，−8 d～−7 d；Cy 25 mg·kg^−1^·d^−1^，−5 d～−2 d；Flu 30 mg·m^−2^·d^−1^，−6 d～−2 d；rATG 2.5 mg·kg^−1^·d^−1^，−5 d～−2 d。

所有患者采用环孢素A+短程甲氨蝶呤+霉酚酸酯方案进行GVHD预防。

三、定义及疗效评估

PGF定义为移植后28 d仍存在持续2周以上两系或三系血细胞减少（中性粒细胞计数<0.5×10^9^/L、血小板计数<20×10^9^/L或血小板输注依赖、HGB<70 g/L或红细胞输注依赖），嵌合率完全供者型，排除复发、严重GVHD、感染以及药物相关骨髓抑制[23]。PGF分为原发性和继发性，继发性PGF是指获得造血重建后出现至少两系血细胞减少。原发性PT定义为移植后60 d嵌合度为完全供者型，但血小板计数仍<20×10^9^/L或血小板输注依赖，而粒系（ANC>0.5×10^9^/L且脱离G-CSF）及红系（HGB≥70 g/L且脱离输注）植入良好[2]；继发性PT（sPT）定义为获得血小板植入后再次出现连续7 d血小板计数<20×10^9^/L或血小板依赖输注[2],[24]。

PGF的造血恢复定义为至少2周中性粒细胞计数>0.5×10^9^/L且脱离G-CSF，HGB≥70 g/L且脱离红细胞输注，血小板计数>20×10^9^/L并脱离血小板输注。PT造血恢复定义为至少2周血小板计数>20×10^9^/L且脱离血小板输注。供者纯化CD34^+^细胞输注后完全造血恢复（CHR）定义为输注CD34^+^细胞后180 d内三系均获得造血恢复，部分造血恢复（PR）定义为输注后180 d内获得二系造血恢复；无效（NR）定义为输注后180 d内未获得二系造血恢复。

四、供者纯化CD34^+^细胞的采集和输注

原供者采用G-CSF 5 µg/kg进行动员，动员至第4日时采集外周血干细胞。应用CliniMACS Plus系统对采集的造血干细胞进行CD34^+^细胞分选，流式细胞术测定CD34^+^细胞数，活细胞染料DRAQ测定分选后细胞活性。输注CD34^+^细胞数为1×10^6^/kg～1×10^8^/kg。分选后的细胞存于4 °C冰箱，于24 h内输注完毕，输注前可予葡萄糖酸钙注射液抗过敏。输注前后患者原有GVHD预防方案维持不变。

五、统计学处理及随访

所有患者均通过门诊或电话随访至2024年3月31日，统计患者造血恢复情况，评估GVHD发生情况及总生存（OS）期。OS期为输注纯化供者CD34^+^细胞到随访截止或死亡时间。计量资料用“均数±标准差”或中位数（范围）表示。*P*<0.05为差异有统计学意义。

## 结果

一、患者一般情况

11例AA患者中男5例，女6例，中位年龄20（11～56）岁。SAA 8例，TD-NSAA 3例。诊断AA至移植的间隔为6（0.58～16）年。所有11例患者移植模式均为单倍体移植，其中例4因原发性PGF行二次单倍体移植。预处理方案：Bu-Flu/Cy+ATG 7例，Bu-Cy+ATG 4例；移植物：骨髓+外周血干细胞10例，外周血干细胞1例；输注MNC中位数为10.92（9.51～17.35）×10^8^/kg，CD34^+^细胞中位数为3.46（1.44～9.57）×10^6^/kg。11例患者均获得粒细胞植入，中位植入时间为12（10～23）d；4例患者未获得血小板植入，中位植入时间为15（7～21）d。11例患者有9例发生急性GVHD，8例发生CMV血症，3例发生EB病毒感染，3例发生出血性膀胱炎，2例并发新型冠状病毒感染。11例患者的一般资料详见表1，造血重建及移植相关合并症发生情况见表2。

**表1:** 11例单倍体造血干细胞移植后造血重建不良（PHR）患者的一般资料

例号	性别	年龄（岁）	原发病	诊断至移植时间（年）	移植前铁蛋白（µg/L）	供者	供受者血型	移植前HLA	移植前DSA	预处理
1	女	31	SAA-Ⅱ	16	8 017	父亲	B+供AB+	Ⅰ类MFI 6842	（B50:01）1041	Bu-Cy+rATG
2	男	29	SAA-Ⅱ	1.25	770	母亲	O+供O+	阴性	阴性	Bu-Flu-Cy+rATG
3	女	33	TD-NSAA	10	358	父	A+供A+	阴性	阴性	Bu-Cy+rATG
4	女	14	SAA	2	879	第一次父亲，第二次母亲	AB+供A+	阴性	阴性	Bu-Flu-Cy+rATG
5	男	20	SAA	14	11 683	父亲	A+供A+	阴性	阴性	Bu-Flu-Cy+rATG
6	女	12	SAA	4	1 838	父亲	O+供B+	阴性	阴性	Bu-Cy+rATG
7	女	11	TD-SAA	7	1 517	父亲	O+供B+	阴性	阴性	Bu-Cy+rATG
8	男	56	VSAA	0.58	8 013	儿子	A+供O+	Ⅱ类MFI 512	阴性	Bu-Flu-Cy+rATG
9	女	11	SAA-Ⅱ	4	566	父亲	B+供O+	阴性	阴性	Bu-Flu-Cy+rATG
10	男	11	TD-NSAA	6	1 104	父亲	AB+供A+	阴性	阴性	Bu-Flu-Cy+rATG
11	男	35	SAA-Ⅱ	8	2 423	母亲	B+供B+	阴性	阴性	Bu-Flu-Cy+rATG

**注** SAA：重型再生障碍性贫血；TD-NSAA：输血依赖的非重型再生障碍性贫血；Bu：白消安；Cy：环磷酰胺；Flu：氟达拉滨；rATG：兔抗人胸腺细胞免疫球蛋白

**表2:** 11例单倍体造血干细胞移植患者的造血重建及移植相关合并症发生情况

例号	移植物	MNC输注量（×10^8^/L）	CD34^+^细胞输注量（×10^6^/L）	粒细胞植活时间（d）	血小板植活时间（d）	移植后合并症
1	BM+PB	14.16	1.61	12	21	aGVHD（皮肤、肝脏、消化道），CMV血症，出血性膀胱炎
2	BM+PB	17.35	3.54	12	未植活	aGVHD（皮肤），CMV血症
3	BM+PB	11.70	2.60	11	未植活	CMV血症，EBV血症，PTLD
4	PB	9.89	3.46	23	未植活	二次移植后反复感染，aGVHD（肠道），出血性膀胱炎，多浆膜腔积液
5	BM+PB	10.82	5.66	13	10	aGVHD（皮肤），CMV血症
6	BM+PB	9.80	9.57	10	10	aGVHD（皮肤），CMV血症
7	BM+PB	11.42	5.26	12	15	新型冠状病毒感染
8	BM+PB	10.92	2.09	16	未植活	aGVHD（皮肤），重症新型冠状病毒感染，菌血症
9	BM+PB	15.71	3.07	11	7	aGVHD（皮肤），CMV血症，菌血症
10	BM+PB	10.44	5.90	13	15	aGVHD（皮肤），CMV血症，EB病毒脑炎，重症肺炎
11	BM+PB	9.51	1.436	10	21	aGVHD（皮肤、肠道），出血性膀胱炎，EBV-PTLD，CMV血症

**注** BM：骨髓；PB：外周血干细胞；aGVHD：急性移植物抗宿主反应病；MNC：单个核细胞；PTLD：移植后淋巴增殖性疾病；EBV：EB病毒；CMV：巨细胞病毒

二、造血不良及CD34^+^细胞输注情况

11例PHR患者中，2例为PT患者，1例为原发性PGF，8例为继发性PGF。诊断PHR的中位时间为110（60～330）d。诊断PGF或PT后，患者均接受糖皮质激素、TPO-RA等多种促造血药物治疗。诊断PHR到输注CD34^+^细胞的中位间隔时间为101（24～325）d，移植到输注CD34^+^造血干细胞的中位间隔时间为194（125～456）d。CD34^+^细胞中位输注量为3.37（1.03～16.8）×10^6^/kg。输注时所有患者均处于基础免疫抑制剂治疗中，较输注前无改变。输注CD34^+^细胞时有1例患者合并口腔及皮肤轻度慢性GVHD，有1例患者合并多浆膜腔积液、心功能不全及新型冠状病毒感染，余患者均无活动性GVHD及感染。输注过程顺利，输注后有2例患者发生皮肤轻度慢性GVHD，原有慢性GVHD患者输注CD34^+^细胞后GVHD稳定。详见表3。

**表3:** 11例单倍体造血干细胞移植后患者造血重建不良（PHR）患者CD34^+^细胞输注相关资料

例号	PHR类型	移植至诊断PHR间隔（d）	PHR合并治疗	诊断PHR至CD34^+^细胞输注间隔（d）	输注量（×10^6^/kg）	输注时GVHD预防	输注时合并症	回输后合并症
1	sPGF	63	维甲酸、TPO-RA、达那唑、G-CSF	107	1.03	CsA	无	cGVHD（皮肤）
2	PT	60	TPO-RA、GC、NAC	101	2.41	CsA	无	无
3	sPGF	64	TPO-RA、NAC、GC、TU、G-CSF	122	1.71	CsA	无	无
4	sPGF	62	GC、TU、NAC、TPO-RA、G-CSF	63	1.91	西罗莫司+地塞米松	多浆膜腔积液、心功能不全、新型冠状病毒感染	多浆膜腔积液、心功能不全、新型冠状病毒感染、TMA、HC
5	sPGF	120	达那唑、TPO-RA、NAC、G-CSF	74	7.99	CsA	无	无
6	sPGF	330	IVIG、GC、TPO-RA	126	16.80	CsA+泼尼松	无	无
7	sPGF	180	TU、阿伐曲泊帕、GC、IVIG、G-CSF	34	8.88	西罗莫司	无	无
8	pPGF	91	TU、NAC、TPO-RA、GC、G-CSF	325	2.69	CsA+MMF+泼尼松	cGVHD（口腔、皮肤）	cGVHD未加重
9	sPGF	110	G-CSF、TPO-RA、NAC、TU、MSC	92	5.36	CsA	无	cGVHD（皮肤轻度）
10	sPGF	210	MSC、NAC、TPO-RA、GC	135	7.16	CsA	无	无
11	PT	137	GC、TU、NAC、TPO-RA	24	3.37	CsA	无	无

**注** sPGF：继发性植入功能不良；pPGF：原发性植入功能不良；PT：持续性血小板减少；cGVHD：慢性移植物抗宿主病；TPO-RA：血小板生成素受体激动剂；GC：糖皮质激素；IVIG：静脉注射免疫球蛋白；NAC：乙酰半胱氨酸；TU：十一酸睾酮；MSC：间充质干细胞；MMF：霉酚酸酯；CsA：环孢素A；TMA：血栓性微血管病；HC：出血性膀胱炎

三、CD34^+^细胞输注疗效及预后

以输注CD34^+^细胞后180 d为观察点进行疗效评估，2例PT患者分别在CD34^+^细胞输注后22 d和13d获得血小板CR；9例PGF患者中，6例获得CHR，ANC中位恢复时间为19（8～158）d，HGB中位恢复时间为32.5（13～158）d，血小板中位恢复时间31.5（7～171）d。例9输注2次供者纯化CD34^+^细胞，首次输注后8 d获得粒细胞造血恢复，但未脱离红细胞及血小板输注；在首次输注后105 d进行第二次纯化CD34^+^细胞输注，在第二次输注后7 d脱离血小板输注，50 d脱离红细胞输注。1例（例6）评估为PR，2例评估为NR，其中例10在CD34^+^细胞输注后8个月获得造血恢复。详见表4。中位随访时间为614（153～1 765）d，例4因心功能不全、TMA死亡，其余10例患者均存活。

**表4:** 11例单倍体造血干细胞移植后造血重建不良（PHR）再生障碍性贫血患者纯化供者CD34^+^细胞输注的疗效及随访结局

例号	PHR类型	ANC恢复时间（d）	HGB恢复时间（d）	PLT恢复时间（d）	疗效评估	随访时间（d）	随访结局
1	sPGF	25	19	85	CHR	1 765	存活
2	PT	/	/	22	CHR	1 279	存活
3	sPGF	41	41	39	CHR	913	存活
4	sPGF	/	/	/	NR	153	死亡（心功能不全、血栓性微血管病）
5	sPGF	13	24	24	CHR	605	存活
6	sPGF	160	172	286	PR	837	存活
7	sPGF	10	13	10	CHR	590	存活
8	pPGF	158	158	171	CHR	731	存活
9	sPGF	8	50（二次输注后）	7（二次输注后）	CHR	527	存活
10	sPGF	255	192	235	NR	614	存活
11	PT	/	/	13	CHR	242	存活

**注** sPGF：继发性植入功能不良；PT：持续性血小板减少；pPGF：原发性植入功能不良；CHR：完全造血恢复；PR：部分造血恢复；NR：无效；/：不适用

## 讨论

AA患者移植后PHR机制目前尚未完全明确，既往研究提示供受者类型、预处理方案、铁过载、CMV再激活、GVHD可能是PGF的危险因素[2]–[4],[12],[23],[25]–[29]。目前“种子、土壤和气候”学说被广泛接受，既往文献提示移植物CD34^+^细胞数量低是PGF的独立危险因素[6]–[7]，即使供者CD34^+^细胞数量和功能正常，移植后骨髓CD34^+^细胞活性氧升高可能导致其功能异常，从而导致PHR[9]，这为供者纯化CD34^+^细胞输注治疗PHR提供了理论基础。

目前AA患者移植后PHR的治疗尚无标准治疗方案，可采用G-CSF、输血、雄激素、糖皮质激素、TPO-RA等，本研究11例患者经上述治疗效果均欠佳。既往关于纯化CD34^+^细胞输注治疗PGF的有效率为58.3％～100％[13]–[22]，大多数为恶性血液疾病，部分报道中含有AA患者，但并未明确AA患者输注CD34^+^细胞的有效率。Fraint等报道了14例儿童移植后PGF的患者，总体有效率79％，其中SAA患者2例，1例CR，1例无效行二次移植后获得造血重建[30]，既往我院的CD34^+^细胞输注治疗8例PGF报道中，包含2例SAA输注后获得了造血重建[22]。本研究11例AA患者移植后发生PHR，输注CD34^+^细胞后72.7％的患者获得完全造血重建，这是首次对AA后PHR输注纯化CD34^+^细胞的研究。

以往研究发现，CD34^+^细胞输注疗效与基础疾病、供受者类型、ABO血型、是否有前期GVHD病史等无关[17]。Cuadrado等[13]报道，62例患者中47例在输注CD34^+^细胞后获得造血恢复，多因素分析发现造血恢复与供受者CMV血清学阴性、不存在活动性感染及供受者性别一致相关。Shapiro等[31]研究发现，CD34^+^细胞数量、移植到输注CD34^+^细胞间隔时间等与疗效均无相关性，而活动性感染是疗效不佳及预后不良的因素。

本研究11例患者均为AA患者，输注时处于基础免疫抑制剂治疗中，输注CD34^+^细胞前后未改变GVHD预防方案。本研究有1例患者合并口腔及皮肤轻度慢性GVHD，输注后慢性GVHD未加重；有2例患者新出现皮肤慢性GVHD（18.2％）。既往文献报告的输注后GVHD发生率不同，Haen等[19]报道输注CD34^+^细胞后急性GVHD发生率为5％，低于Klyuchnikov等[21]和Askaa等[20]报道的结果（分别为19％、22％），推测GVHD的发生率与回输物中CD3^+^ T细胞数量相关。

本研究结果初步表明，纯化CD34^+^细胞输注治疗AA患者单倍体移植后PHR有效且安全。以上结论尚需大样本前瞻性研究加以验证。
